# Antioxidant Effect and Acute Oral Toxicity of Hot Springs

**DOI:** 10.1155/2022/4200824

**Published:** 2022-09-28

**Authors:** Israa J. Hakeem, Gashaw Tadele Zewudie

**Affiliations:** ^1^University of Jeddah, College of Science, Department of Biochemistry, Jeddah, Saudi Arabia; ^2^Department of Chemistry, Mizan-Tepi University, Ethiopia

## Abstract

According to the research, there are many illnesses for which therapeutic mineral hot springs are employed as an alternative. Its physicochemical characteristics have a substantial body of evidence. The in vivo antioxidant effect of Mosul's hot springs in Iraq has been investigated in the current investigation. An experimental design for toxicity, a control group, and a study group were created. In addition, *in vivo* antioxidant effect of the hot springs of Mosul, Iraq, has been studied by the lipid antiperoxidation method with (*p* < 0.05), in vitro by the free radical scavenging method (DPPH) for its complexing capacity of hot springs. In acute oral toxicity in vivo at fixed doses, looking for signs and symptoms of toxicity, there are no signs of intoxication or significant changes in the biochemical analysis (blood count). And, it was discovered that the variances are substantial. The animal was necropsied, and hematological and biochemical parameters were determined, as well as the organs' histological processing at the study's conclusion. It was found that the thermal waters from Mosul, Iraq, are medicinal mineral waters, chlorinated, sodium, and sulfated, nontoxic and have an antioxidant effect. With the help of the research's findings, it is hoped to provide scientific support for knowledge that, when made public, encourages the development of Mosul's hot springs as a safe and environmentally friendly tourist destination. With the results of this research, the parameters were presented with their mean and standard deviation statistics, promoting the ecological and sanitary tourism development of the Mosul hot springs, which will generate more significant income for the population, therefore growth in the region.

## 1. Introduction

Since mineral waters have been a part of our existence since ancient times, understanding its origin and mineralization via an investigation of the formation mechanisms that give birth to this water's source and their interaction with the formations in which it is collected has tremendous value [1]. Such is the case that it was established in 2013 [2, 3], as a natural resource, for the treatment of various diseases. Groundwater and its hydrogeological context are related to physical-chemical and biological geological factors. [4, 5] Within the hydrological cycle, waters with characteristics can be differentiated by the degree of mineralization and/or by their temperature from groundwater, called mineral waters [6]. The intake of natural mineral water containing sulfates leads to gallbladder contraction, probably induced by the release of cholecystokinin [7]. Water toxicity can be determined by applying the 16-day repeated dose oral toxicity test in rodents; this method provides information on the health risk likely to arise from exposure to the test substance by oral administration [8]. This guide is primarily intended for use with rodents, preferably rats.

Some diseases related to free radicals are increased in our environment because the body's antioxidant defences are overcome by oxidative attack due to the work environment or other conditions that give rise to oxidative stress [9]. These disorders are caused by the oxygen's damaging effects, especially those of its free radicals in the mitochondria, which target all the elemental constituents and create various metabolites like malondialdehyde [10]. There are a variety of cases of acute intoxication, which manifested with a pathological clinical picture, after a single exposure to a substance or multiple exposures in a period of 24 hours [11]. For all these reasons, it was decided to study the antioxidant effect and acute toxicity of the hot springs of Mosul, Iraq, determining the antioxidant effect according to the lipid antiperoxidation method, the acute oral toxicity, the concentrations of sodium, potassium, calcium, magnesium, sulfates, chlorides, and other corresponding in the four seasons of the year, to provide scientific support for its use as mineral-medicinal thermal water.

## 2. Materials and Methods

The water sample was collected from the thermal waters from Mosul hot spring at an altitude of 36.361312′, 43.120445′ longitudes. To preserve and avoid decomposition, it was transported to the laboratory at a temperature of 4–8°C. To carry out the analyses, the techniques of the standard methods for the examination of water and wastewater are a joint publication of the American Public Health Association (APHA), 23^rd^ edition [12]. The peristaltic pump is used to convey the liquid sample to the nebulizer system, where it is converted into an aerosol by the action of argon gas before being sent to the ionization zone for ICP-MS analysis [13]. A high-frequency current induces an oscillating magnetic field that creates a plasma from a flow of argon gas. Plasma can reach temperatures as high as 8000 K. The atoms in the sample are ionized under these circumstances. An increasing vacuum interface separates the charged and noncharged particles in the quadrupole filter. The sample's abundance of each tuned mass is determined at the detector. In ion exchange chromatography, the charge characteristics of molecules are used to separate ions and polar molecules for further analysis. Large proteins, tiny nucleotides, and amino acids are all examples of charged molecules that can benefit from this technique. The injectable solution is referred to as a “sample,” and the constituents are referred to as “analytes.” Anions are the analytes in this scenario (chlorides, sulfates).

### 2.1. Determination of Toxicity at Repeated Doses for 16 days by Oral Administration in Rats

20 Wistar rats were used in healthy young adult nulliparous and nonpregnant females commonly used laboratory strains Group 1 control (autoclaved drinking water) consisting of five females and five males and Group 2 study (thermal water of Mosul, Iraq) consisting of five females and five males. The trial was conducted for 16 days and administered for four days. A dose was used for the test sample, 1000 mg/kg body weight. The volume to be administered every four days was adjusted, which depended on the study group's body weight variations. In addition, the limit volume (of aqueous solutions) for this species of 2 ml/100 g of body weight was considered.

Maintenance and Feeding: the experimental animals were adapted to the bacterium's conditions for four days before starting the test; it is distributed individually in polycarbonate boxes type *T*3 (plastic) with grid-bottom metal.

Water and food were sterilized with an autoclave to avoid any contamination. The environment's temperature was conditioned to 23 ± 2.1°C, relative humidity <70%, and photoperiod of 12 hours of light and 12 hours of darkness.

The clinical and pattern signs of behaviors related to the toxicity of the experimental animals were observed daily, as well as changes in the skin, fur, mucous membranes, eyes, hyperventilation, vasodilation, restlessness, and somatic motor activity. Special attention was paid to the possible occurrence of some crucial signs for the study, such as tremor, convulsion, diarrhea, lethargy, irritability, salivation, sleep, and coma.

Daily and four days measurements of water and food consumption were made. Individual body weights were determined the day before the hot springs and drinking water administration, then at the 3, 8, 10, and 15 days that the rehearsal lasts. Hematological and biochemical parameters (blood count, urea, creatinine, TGO, TGP) were determined 16 days after being treated with the sample problem; blood samples were extracted by intracardiac puncture from the previously anesthetized and fasting animals.

On the last day of the experimentation, the animals of the study group and the control group were sacrificed for cervical dislocation after anesthesia. Then, the autopsy was performed where the body surface, cavities, and organs were examined.

#### 2.1.1. Evaluation of the Antioxidant Effect

*(1) Technique for the Detection of Lipid Peroxidation Products (TBARS)*. For this process, the livers of female albino rats were used, weighing 200–220 g, and kept in an acrylic cage, to which balanced food and water were provided ad libitum, stopping feeding them 12 hours before the experiment. The animals were divided into two groups [14]. The five rats in the control group were given 5 mL/kg of body weight of water suitable for human consumption, while 15 rats, divided into groups of 5 rats, were given hot springs at a rate of 3, 5, and 9 mL/kg of weight, respectively. Then, 1 g of the liver was separated and homogenized in 10 volumes of 0.154 M KCl, and subsequently, the production of the malondialdehyde-thiobarbituric acid complex (MDA-TBA) was measured at 535 *µ*m in a UV–visible spectrophotometer [15].

During the first 30 minutes of the first four hours after injection and then every day for the next 16 days of the experimental study, individual animals were watched for any indications or symptoms of toxicity. The evaluation's goal was to determine the cause of death and the exact moment that poisoning signs and symptoms, weighing 28 to 32 g. They were stopped feeding 12 h before the experiment.

Five mice for the control group were dosed with a dose of 20 mL/kg body weight of 0.9% sodium chloride. The study group consisted of 5 groups of 5 mice each. As we worked with five different doses: 10, 20, 30, 40, and 50 mL/kg, each group received only one of them, respectively. (7). The animals were observed individually during the first 30 minutes of the 4 hours following administration and then daily until the 16 days of the experimental trial, looking for signs and symptoms of toxicity. The evaluation was aimed at determining death and time of occurrence of signs and symptoms of toxicity, including its onset and duration, as well as changes in the skin, mucous membranes, and eyes, in the respiratory, circulatory, central nervous, and autonomic systems, in somatomotor effect and behavior. Special attention was paid to the potential occurrence of seizures, salivation, diarrhea, lethargy, drowsiness, and coma. At the end of the period, for assessing any toxicity-related symptoms and indicators, the animals [16] were sacrificed and subjected to necropsy in which the macroscopic pathological changes of organs and tissues were observed, mainly in the heart, lung, liver, spleen, and kidney. Subsequently, a histopathological examination of the liver and kidney was performed.

The description of variables is expressed in means and standard deviation; statistical comparison of groups, using one-way ANOVA followed by Tukey's post hoc test, was considered significant with *p* < 0.05 at the 95% confidence interval. The statistical program SPSS version 17 was used.

## 3. Results

**Figure 1:**
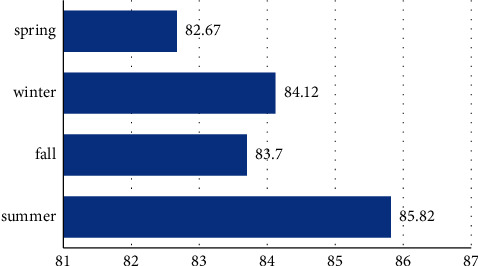
Annual behavior of metal concentrations.

Table 1 shows the results of the analytical determinations of total metals (cations), during the four seasons of the year 2022, of the thermal water of Mosul.  Table 2 and Figure 1 show the descriptive statistics of the metal results, and Tables 3 and 4 show the average values of cations and anions in (mg/L), meq/L, and % meq.

Table 5 describes the results of the observation of the experimental animals for 16 days of testing for antioxidant study.

### 3.1. Determination of Toxicity at Repeated Doses for 16 days by Oral Administration in Rats

#### 3.1.1. Clinical Observations

**Figure 2:**
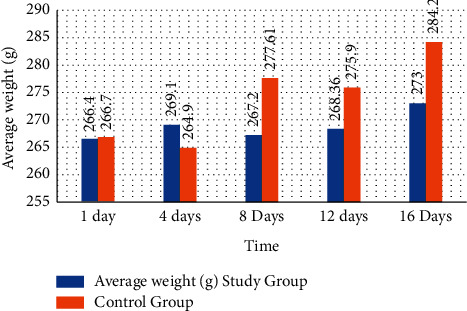
Variation of the body weight of the Wistar rats that received the thermal water of Mosul orally for 16 days.

The observations of the clinical signs of the animals were made daily; fundamentally, the physical state, behavior in the nasal and ocular mucous membranes, secretions, or alterations related to toxicity were sought [17]. Palpation of the abdomen was performed without finding changes, and special attention was paid to the possible occurrence of signs such as tremors, convulsions, diarrhea, lethargy, salivation, sleep, and coma. And at the end of the experimentation, no changes or alterations in the clinical signs were observed. They considered the expected behavior of the animals of said species. The animals were weighed at 1, 4, 8, 12, and 16 days. The results show that the tendency to increase body weight was constant during the study in both groups (Figure 2).

**Figure 3:**
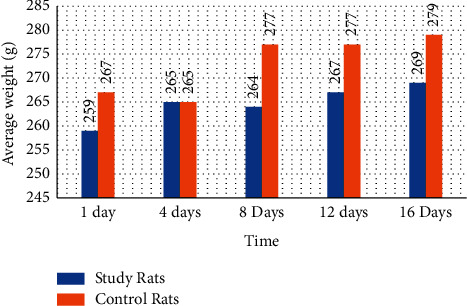
Behavior of the mean weight values of Wistar rats from the control and study groups during the 16 days of the study.

In Figure 3, it can be seen that when comparing the study group and the control group with time, there is no significant difference; no effect of the groups and time on the weights of Wistar rats was found.

### 3.2. Clinical Laboratory Tests

Hematological and blood chemistry determinations were carried out 16 days after gastric gold administration of the hot springs. Blood collection was performed by cardiac puncture after fasting for 12 h of the animals; safety vials were collected. In both cases, the parameters were presented with their mean and standard deviation statistics, which groups established. With the statistical treatment, no changes or alterations in blood formula were evident. In the anatomopathological study, at the end of the treatment, there was no evidence of the death of any animal, so euthanasia was performed on the 20 experimental animals in which ether was previously applied [18]. Then, cervical dislocation was performed in order to provide the necessary macroscopic observations for later microscopic research and then proceeded to the extraction and weighing of the following organs: heart, lungs, kidneys, spleen, and liver. No significant difference was observed concerning the weights of the organs; in addition, in the microscopic histological study that was carried out on the lung, heart, spleen, and liver, no macroscopic or microscopic alterations were attributable to the sample were found under study. There is no significant difference in the weights of the organs of the control group concerning the study group. There was no animal death during the study, so all were sacrificed at the end of the investigation.

## 4. Discussion

The studies carried out for the chemical analysis of the hot springs of Mosul, Iraq, reported the presence of 0.2871 g sodium sulfate, 3.1955 g sodium chloride, etc. In 2018, the Governorate of Mosul requested the Water Research Laboratory for the chemical analysis of four water samples [19]. The test report reports that the following potassium was 57.69 mg/L, and sodium was 1350 mg/L. Al-Shahri [20] states that “the thermal and mineral waters of Mosul are sodium chloride and sulfated.” Hashim, in 2020, [21] in his study of the chemical and toxicological evaluation of lithium, found 15.95 mg/L of said element in the thermal water. Hashim [21] affirms that the thermal water of Mosul presents sodium 1682.31 mg/L, chloride 1962.31 mg/L, and sulfate 861.91 mg/L; however, in the study that was carried out, sodium 1869.63 mg/L, sulfate 7796.30 mg/L, potassium 75.06 mg/L, chloride 1610.62 mg/L, and by concerning previous studies, sodium concentrations increased by 27% and potassium by 24% over time. However, the concentrations of sulfates decreased by 11% and chlorides by 20%, decreasing as time went by, probably due to the degradation of volcanic rocks, among others. Table 1 shows the results of some total metals above the detection limits (DL) of the 52 metals analyzed during the four seasons of the year 2022, highlighting some metals of toxicological interest: arsenic, cadmium, mercury, lead, sodium, and potassium but when making comparisons of the values in different seasons of the year, there is no significant difference between them.

Table 2 shows that the mean of the metals in the summer season is higher. The mean values of the metals corresponding to the spring are lower and probably vary with the telluric movements, degradation of the internal rocks, or, failing that, with the rains that drag minerals to the water table; however, these differences are not significant *p* > 0.005. Figure 1 shows that the averages of the concentrations tend to decrease. However, this difference is not statistically significant or the variation between the year's seasons with *p* > 0.005.

In addition, Table 4 shows the values of the concentrations of the chemical elements that indicate the classification of the waters and their chemical equivalent of biochemical and therapeutic interest, considering that the concentration is more significant than the concentration of 1 g/L or 80 meq/L has therapeutic applicability. When an ion is greater than 20 mEq/L, this gives the water its name. According to the predominant ionic composition, mineral waters are classified as chlorinated, sulfated, and sodium waters.

In Table 5, it is observed that only half an hour after the administration of the 20 mL/kg dose, the animals presented clinical signs, slight drowsiness, and grouping in the center of the cage. Subsequently, signs of recovery, normal postural reflex, normal grooming habits, and consumption of food and water were observed until day 16. However, in Figure 1 as demonstrated by the multivariate statistical model of the influence of the control group, study, and time on weights over the 16 days of research, this difference is not statistically significant. In addition to the aforementioned statement, it may be inferred that, even if there are no overt clinical changes, it is possible that the thermal waters would be having a purgative impact on rats due to the loss in weight. There was no evidence of a group or time effect on weights. You can see the difference between the weight averages between the first and second dose times, where the study group's values start to slow down weight gain while the control group's values continue to gain weight over the course of the study.

On the other hand, the histological sections of the different organs (liver, kidney, and heart) of the mouse presented alterations typical of foreign agents, which when entering the organism modifies the cellular physiology, thus microvascularization was observed in hepatocytes, altered bile ducts, and glomerulonephritis to a lesser extent, while the heart remained normal. These alterations regenerate, and it demonstrates that at the dose given, they may not typically entail permanent harm, so it would be advisable to carry out new studies that evaluate chronic toxicity. NaCl 0.9% 5 mL/kg at 0.0527TBARS hm, for ATP3 0.0430TBARS hm; for ATP5 0.0418TBARS hm, and for ATP9 0.039TBARS hm observed. The results show that there is a significant difference between the results of the control group (NaCl 0.9% 5 mL/kg of weight) and those of the study groups (hot springs 3, 5, and 9 mL/kg of weight, respectively), showing that there is an inversely proportional relationship between the dose administered and the absorbance reading reached (lipid peroxidation). This demonstrates the antioxidant activity of the studied waters.

In his study, Eixarch, magnesium sulfate can relieve oxidative stress and reduce inflammatory cytokines in the placenta of rats from intrahepatic cholestasis to the pregnancy model. They concluded that MgSO_4_ had a beneficial effect on improving the growth of offspring in the rat model of PCI [22]. The protective effect of MgSO_4_ in relieving oxidative damage and the inflammatory response in the placenta may play an essential role in the process. MgSO_4_ can improve the placenta's function. In our study, magnesium and sulfate were determined in considerable concentrations that would probably produce a protective effect against oxidative stress. As far as toxicity is concerned, the study was carried out to determine the toxicity of the thermal waters of Mosul, Iraq, administered to Wistar rats by orogastric route once a day for 16 days.

Biochemical parameters such as creatinine are considered markers of kidney damage which are directly related to body mass; it is evident that the rats in the study group (100 mg/kg) decreased creatinine values; these values coincide with the loss of body weight during the second dose of treatment. Agree with the values found in our study that indicate there are no signs of intoxication.

## 5. Conclusion

The components and chemical concentrations of the thermal water of Mosul, Iraq-Puno, were determined in the year's four seasons, being an average of the highest concentrations, Na^+^ 1682.31 mg/L, Cl^−^, 864.32 mg/L, SO_4_^2−^ 801.61 mg/L and K^+^, 80.21 mg/L. The chemical components of the thermal water of Mosul, Iraq, have the highest average concentration in summer (86.31 mg/L) and the lowest average concentration (83.07 mg/L) in spring. The thermal water of Mosul, Iraq, is not toxic in Wistar rats as it is administered orally for 16 days.

## Figures and Tables

**Table 1:** Total metals of the thermal water of Mosul during the four seasons.

Total metals	DL	M1	M2	M3	M4
		Summer	Fall	Spring	Winter
Aluminium (mg/L)	0.0011	0.0396	BDL	BDL	BDL
Antimony (mg/L)	0.006237	0.00704	0.006237	0.005687	0.005775
Arsenic (mg/L)	0.000033	0.053108	0.056221	0.052426	0.054472
Barium (mg/L)	0.00011	0.04202	0.04213	0.04191	0.04191
Boron (mg/L)	0.0022	50.9509	50.1479	51.7979	51.2402
Cadmium (mg/L)	0.000011	BDL	BDL	BDL	BDL
Calcium (mg/L)	0.0033	254.9041	247.8619	244.233	243.1836
Caesium (mg/L)	0.00011	2.83426	2.92952	2.87221	2.95152
Cobalt (mg/L)	0.000011	0.000429	0.000418	0.000451	#Value!
Strontium (mg/L)	0.00022	6.73464	6.34711	6.41311	6.29211
Germanium (mg/L)	0.00022	0.01221	0.01188	0.01243	0.01177
Lithium (mg/L)	0.00011	24.55926	24.28162	24.09143	22.99561
Magnesium (mg/L)	0.0011	57.8523	59.0073	58.2835	59.2636
Manganese (mg/L)	0.000033	0.224235	0.207306	0.202367	0.101167
Mercury (mg/L)	1.1	BDL	BDL	BDL	BDL
Molybdenum (mg/L)	0.000022	0.004972	0.004422	0.004411	0.004323
Lead (mg/L)	0.00022	BDL	BDL	BDL	BDL
Potassium (mg/L)	0.044	86.02	83.149	78.562	81.939
Rubidium (mg/L)	0.00033	0.30635	0.28732	0.28677	0.28204
Selenium (mg/L)	0.00044	0.00275	0.00242	0.00319	0.00231
Silica (mg/L)	0.099	49.203	49.302	49.61	51.205
Silica (mg/L)	0.044	23.001	23.045	23.188	23.936
Sodium (mg/L)	0.0066	1,803.19	1,72,666	1,762.05	1,769.70
Thallium (mg/L)	0.000022	0.000363	0.000352	0.000352	0.000286
Titanium (mg/L)	0.00022	0.02618	0.02497	0.02387	0.0242

^*∗*^BDL: below detectable limit.

**Table 2:** Descriptive data of the total metals found in Mosul's thermal water throughout the course of the four seasons.

Station	Statistical	*P*
Half	85.82	
Summer medium	0.0483	
Minimum	0	
Maximum	1639.26	
Half	82.67	0.99^*∗*^
Medium spring	0.0511	
Minimum	0	
Maximum	1569.69	
Half	83.7	
Medium autumn	0.0477	
Minimum	0	
Maximum	1601.86	
Half	84.12	
Medium winter	0.0495	
Minimum	0	
Maximum	1608.82	

**Table 3:** The four stations' average cation values.

Cation	mg/L	meq/L (%)
Sodium	1,604.91	69.78 (77.1%)
Potassium	74.925	1.94 (2.14%)
Lithium	21.8	3.15 (3.47%)
Calcium	225.04	11.23 (12.41%)
Magnesium	53.27	4.39 (4.85%)
Total	1,979.94	90.49 (100%)

**Table 4:** The four stations' average anion values.

Anions	mg/L	meq/L (%)
Chloride	1,829.83	51.62 (72%)
Bicarbonate	253.63	4.16 (5.8%)
Nitrate	0.04	0 (0%)
Sulfate	763.97	15.91 (22.18%)
Total	2,847.47	71.69 (100%)

**Table 5:** Result of the observation of experimental animals for 16 days at a dose of 20 mL/kg.

Observation period	Clinical signs
Half an hour after administration	Slight drowsiness and grouping in the center of the cage
From 3 hours after administration	Signs of recovery
From 4 hours after administration	Normal postural reflex, normal grooming habits, and consumption of food and water
Second day	Intermittent bunching at cage ends
From the third day, until the end of the study	No signs of toxicity were observed, and the animals were normal in their behavior and lifestyle

## Data Availability

The data underlying the results presented in the study are available within the manuscript.
